# Laminated Wallboard Panels Made with Cellulose Nanofibrils as a Binder: Production and Properties

**DOI:** 10.3390/ma13061303

**Published:** 2020-03-13

**Authors:** Islam Hafez, Mehdi Tajvidi

**Affiliations:** 1 Laboratory of Renewable Nanomaterials, School of Forest Resources, University of Maine, 5755 Nutting Hall, Orono, ME 04469, USA; mehdi.tajvidi@maine.edu; 2 Advanced Structures and Composites Center, University of Maine, 35 Flagstaff Road, Orono, ME 04469, USA

**Keywords:** cellulose nanofibrils, gypsum panels replacement, fire resistance, green building

## Abstract

This study explored the development and characterization of laminated wallboard panels using renewable materials for building applications. The panels are based on cellulose nanofibrils (CNFs) as a binder and wood particles. Other additives included FiberLean^TM^ (microfibrillated cellulose/calcium carbonate composite), starch and fire retardant (boric acid/borax). These panels are also intended to address the environmental concerns of commercial gypsum boards. The manufacturing of the panels is via a wet-based process; hence no initial drying was required to remove the water from the CNF. It was found that the dosage of CNF (and/or FiberLean^TM^) binder and the addition of starch had the largest impact upon the quality of the final product. The addition of starch was found more favorable in the presence of FiberLean^TM^. The fire retardancy was induced by adding boric acid/borax (1:1). The burning test revealed that the panels treated with the fire retardant exhibited excellent burning properties comparable to that of gypsum board (inherently fire resistant). Interestingly, the addition of the boric acid/borax also appeared to increase the retention of starch in the system, leading to favorable mechanical properties.

## 1. Introduction

Currently, the manufacturing of wood-based panels is heavily relying on petroleum-based adhesives [1]. These adhesives, despite their low cost, have severe impacts on the environment and human health. Particleboard manufacturers primarily use phenol- and urea-formaldehyde, which are highly toxic for use in households due to formaldehyde emissions. Using renewable and bio-based adhesives for wood-based panels does not often come without challenges, most of which are related to the commercialization of these bio-based adhesives [2].

Of the different types of renewable adhesives, cellulose nanofibrils (CNF) have been found promising for their adhesion capability [3] and potential commercialization [4]. Typically, cellulose fibers are made of an assembly of macrofibrils, which are composed of even smaller elements called microfibrils [5]. The fibrils are composed of highly-ordered regions (crystalline domains) and less-ordered regions (amorphous domains) [6]. At the molecular level, cellulose is a linear homopolymer of glucose units bonded covalently via 1, 4-β-glycosidic linkages [7]. The linear chains of cellulose molecules are bonded together through a network of intra- and intermolecular hydrogen bonds [8]. The network of hydrogen bonds gives the cellulose fibrils their high axial stiffness and renders this cellulose a relatively stable polymer [6]. The parallel stacking of multiple cellulose chains is supported through van der Waals interactions. CNF, first developed by Turbak et al. (1983) and Herrick et al. (1983) [9,10], are high aspect ratio nano-elements, mostly produced by high-pressure homogenization or an ultra-fine grinding of cellulosic materials [11]. 

The high surface area, induced by the nanoscale size, allows a high degree of adhesion [8], due to the increasingly exposed hydroxyl groups. CNF are often prepared in a form of suspension at a solid content of 1–5 wt%. A major challenge of using CNF in many applications is the removal of the existing water. There are several available methods that can be applied to remove the water from nanocellulose [12]. Centrifugation is one way to increase the solid content, but this method does not remove a significant amount of water. Other more efficient methods could be used, such as conventional thermal drying, freeze-drying, or supercritical drying [13]. These methods, however, are either energy-intensive (e.g., thermal drying) or not applicable at the large-scale (e.g., freeze-drying). In addition, the drying of cellulose nanomaterials often leads to the loss of nanoscale size, diminishing the effectiveness of the particles in its target applications [13]. Recent developments in the dewatering of nanocellulose highlighted the possibility to remove the water at no energy inputs [3,14,15,16]. These developments showed that water can be removed easily from nanocellulose when mixed with wood particles by applying pressure at room temperature, and several products have been produced using this methodology.

For example, a study by Shivyari et al. (2016) [17] examined the development of a novel laminate structure made of sheets of paper bonded together through CNF. The laminate system showed no severe delamination within the laminates, and the novel system met (or exceeded) the properties of the currently used, reinforced polymer composite materials. In different studies conducted by Leng et al. (2017, 2016) and Amini et al. (2017), CNF was applied as the sole binder to produce medium- and low-density particleboard [3,14,15]. On average, the low-density grades met the industrial requirement for particleboard manufacturing. More recently, CNF was successfully hybridized with a fungal mycelium-based resin to produce composite panels [18]. These studies paved the way for further investigations on the development of building products based on CNF as a binder. The current cost of CNF production is $50 per pound of dry mass. This price tag is based on the currently available pilot-scale production at the University of Maine. With the expected commercialization of nanocellulose, the price tag is expected to further decrease to $2–$5 per pound [4]. In an attempt to decrease the current prices of cellulose nano-(or micro-) fibrils, companies such as FiberLean^®^ Technologies, UK add minerals (e.g., calcium carbonate) to cellulose pulp during the grinding. This step helps reduce the grinding energy, and hence reduces the cost of the product. 

One of the building products that could be addressed, due to environmental and mechanical concerns, is gypsum board. Gypsum board, also known as drywall, is one of the most widely used components in building systems. This product consists of a core of gypsum between paper surfacing. These boards are passively fire retardant [19], and are available at low cost. Despite its availability and low-cost, gypsum board suffers from several major drawbacks. First, during constructions and demolitions, gypsum boards are known to generate a large amount of gypsum and silica dust, that is known to be harmful to the lungs. It has been estimated that a typical single-family house (~2000 sq. ft.) generates a metric ton of gypsum board waste, and a 50,000 sq. ft. office building generates ~16 metric tons of waste [20]. According to the Centers for Disease Control and Prevention (CDC), drywall sanders are exposed to as much as 10 times the permissible exposure limit of 15 mg/m^3^ for total dust set by the Occupational Safety and Health Administration (OSHA) [21]. Second, gypsum board is known to be brittle [22], which adds a limitation to its applications, and increases waste during construction. Questions have also been raised about the disposal of gypsum board in landfills. The sulfate component of the gypsum board can be degraded by sulfate-reducing bacteria (anerobic microorganisms), which produce toxic levels of hydrogen sulfide gas [20,23]. This gas is flammable and hazardous, and could lead to severe health problems. This indicates a need to explore eco- and health-friendly alternatives to gypsum boards. Until recently, there have been no reliable attempts to seek environmentally friendly alternatives to gypsum boards.

This paper addresses the problems linked to the gypsum board by developing and characterizing laminated wallboard panels based on CNF as a binder. In this study, three types of binders (CNF, CNF and FiberLean^TM^ mix, and FiberLean^TM^) were tested. Different binder fractions and target densities were also tested.

The prepared panels (initially without fire retardant) using various types of CNF-based binders and loadings were evaluated for their mechanical properties, thickness swelling and water absorption in the presence and absence of starch. Following this, the effect of boric acid/borax on fire retardancy and mechanical properties was evaluated. The interaction between the various components and their influence on the bonding was also assessed.

## 2. Materials and Methods

### 2.1. Materials

Cellulose nanofibrils (CNF) of 3 wt.% solid content was produced from mechanically refined wood pulp at the University of Maine’s Process Development Center (PDC). FiberLean^TM^ (microfibrillated cellulose and calcium carbonate CaCO_3_, 50:50) was kindly supplied by FiberLean^®^ Technologies (Par, UK). Finely divided southern pine particles were obtained from Georgia Pacific (Thomson, GA, USA). Hydroxyethylated corn starch (Ethylex^®^ 2025) was purchased from Tate and Lyle (Dayton, OH, USA). Boric acid, sodium tetraborate decahydrate (Borax), and potassium iodide were purchased from Fisher Scientific, Inc (Waltham, MA, USA). Paper laminae from recycled, old corrugated containers (OCC) of 200 g per square meter (gsm) were also provided by the Process Development Center of the University of Maine (Orono, ME, USA).

### 2.2. Panel Formation

**Figure 1:**
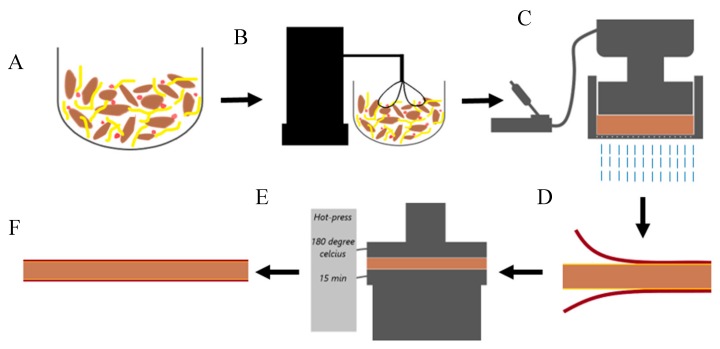
Schematic diagram of the panel formation process: (**A**) all components are placed in one container, (**B**) mixing wood particles, binder and additives, (**C**) contact dewatering via hydraulic pressure, (**D**) introducing two laminae of paper bonded to the core through a layer of CNF, (**E**) hot-pressing and (**F**) final product.

Figure 1 shows the panel formation process. The main components needed to form a structurally-sound panel were wood particles and CNF-based binder. The dry mass of the components was determined based on the target density (0.6 or 0.5 g/cm^3^) and the volume of the panel. Three binder levels were tested: 10 wt%, 15 wt% and 20 wt%. The binder itself was formulated as follows: pure CNF, CNF:FiberLean^TM^ 50:50, and pure FiberLean^TM^. Starch and boric acid/borax (1:1) were added at 7.6 wt.% and 5 wt.%, respectively, of the total dry mass of the panel (wood particles plus binder).

No initial drying was performed on any of the components. In other words, CNF and FiberLean^TM^ (3% and 3.6% solid content, respectively) were used in their wet form. The wood particles and the binder (and the additives) were mixed using a stand mixer for 3 min at low to medium speed, until a homogeneous mixture was obtained. The mixture was then placed into a metal frame (12.6 × 12.6 cm) with a wire mesh at the bottom to prepare for the subsequent contact dewatering. Contact dewatering is a term coined at the University of Maine, which describes the process by which water is removed from CNF without employing heat. The process entails the removal of water from CNF when mixed with wood particles upon cold pressing. It is worth noting that CNF was not drained with water during this process, even despite the relatively larger opening size of the wire mesh (40 mesh) relative to CNF, and this was discussed in detail by Amini et al. (2019) [24].

A Dake^®^ manual hydraulic pump (Grand Haven, MI, USA) was used to press the mat at a pressure of 3.7 MPa to remove the water. After dewatering, the wet-basis moisture content was reduced from approximately 80% to 40%. Prior to hot-pressing, two laminae of paper were added onto the top and bottom surface of the mat, and were bonded by a layer of 2 wt.%-solid CNF (0.22 g dry mass CNF per one surface) applied by a glass rod. This paper laminate had a thickness of 0.29 mm, and with a tensile strength and tensile modulus of 41.8 MPa and 2.20 GPa (machine direction), respectively. The assembly was hot-pressed using a Carver press machine (Wabash, IN, USA) at 180 °C and 1.15 MPa for 15 min to densify and consolidate the laminated panel. The panels were cooled down and conditioned for at least 48 h to prepare for the subsequent testing.

Prior to adding starch to the formulation, it must be cooked and ‘solubilized’. To cook the starch, a 24 wt.% starch slurry was ramped on a hot-plate to a temperature of 88–96 °C. The starch was cooked for 30 min at this temperature, while maintaining sufficient and constant agitation throughout the cooking process. Once the cooking was completed, the solubilized starch was cooled to room temperature and the agitation was halted.

### 2.3. Characterization

Two types of scanning electron microscopes (SEM) were used to examine the various morphologies of CNF, FiberLean^TM^ and the panels at the microscale. To assess the particles of CNF and FiberLean^TM^, freeze-dried samples (~3 wt.% solid content) were analyzed using a Hitachi TM3000 tabletop electron microscope (Tokyo, Japan). This microscope does not require sample coating with a conductive layer of metal prior to imaging. The images were taken at an accelerating voltage of 5 kV and a working distance of 2–3 mm. To examine the panels at the microlevel, a Zeiss NVision 40 microscope (White Plains, NY, USA) was used. This microscope was used to examine the changes occurring in the calcium carbonate particles in the presence and absence of starch in the panels. To this end, a small piece of the panel was placed on a sample holder and coated with gold/palladium (15-nm thick) to enable the imaging of samples. All of the images were taken at a working distance of 4 mm and an accelerating voltage of 4 kV.

The atomic force microscopy (AFM) was used to further examine the morphology of the binder material (CNF and FiberLean^TM^) in their slurry form. AFM imaging was conducted using an ezAFM from NanoMagnetics Instruments, Oxford, UK. A drop of diluted suspension (approximately 0.1 wt.% solid) was placed onto the sample holder and air-dried at room temperature. The imaging was done in dynamic mode (also known as tapping mode) at a scan range of 20 × 20 μm and a scan speed of 2 µm/s.

A laser diffraction technique was used to determine the relative particle size of CNF and FiberLean^TM^. Diluted suspension (0.5 wt%) was circulated in the cell of Malvern Hydro 2000s (Westborough, MA, USA) and the laser hits the particles in the suspension. The scattered light angle and intensity were measured and then transformed into particle size data through an algorithm. The reported particle size represented the diameter of a sphere having the same volume as the particle [25].

The zeta potential (ζ) was evaluated by a Malvern Zetasizer Nano ZS Zen 3600 instrument (Westborough, MA, USA). An initial suspension of 0.5 wt.% was injected into the cell and equilibrated for 120 s. The zeta potential values were obtained from the conversion of the electrophoretic mobility measurements using the Smoluchowsky formula [26,27].

The mechanical performance of the panels was evaluated by the three-point flexural test modified from ASTM C473 [28]. The test was performed using an Instron 5942 testing machine (Norwood, MA, USA) equipped with a load cell of 500 N. In all the flexural testing, the bearing edges were perpendicular to the machine direction of the paper laminae. Commercial gypsum board (CGB) of density of 0.67 g/cm^3^ was used as a reference sample.

Prior to testing, the specimens (110 × 30 × 9.5 mm) were conditioned at a relative humidity of 53% ± 2% and temperature of 23 ± 2 °C for 48 h. Each specimen was supported centrally on the fixed parallel supports (8-cm apart), and a load was applied midway between the supports until failure at a loading rate of 5 mm/min.

The internal bond (tension perpendicular to the surface) was measured by an Instron 5882 testing machine (Norwood, MA, USA) according to ASTM D1037 [29]. This test was used to determine the cohesion of the panel in the direction perpendicular to the plane. Prior to testing, the specimens were conditioned at a relative humidity of 53% ± 2% and temperature of 23 ± 2 °C for 48 h. The test specimens (50-mm square) were bonded between two loading blocks, also 50-mm square, made of aluminum using Surebonder^®^ DT-20M10 hot-melt adhesive (Wauconda, IL, USA). The bond strength between the loading blocks and the hot-melt adhesive should be more than the cohesive strength of the material perpendicular to the plane. The test was conducted at a displacement rate of 20.3 mm/min. The internal bond was calculated according to Equation (1).

(1) $$\text{Internal bond}=\frac{\text{Maximum load}}{\mathrm{Length}\times \mathrm{width}}$$

Thickness swelling and water absorption were measured according to ASTM D1037 [29]. Prior to submerging in water, the mass and dimensions of the specimens were measured. In the first cycle, the specimens (35 × 30 × 9.5 mm) were submerged horizontally for 2 h in water at a temperature of 23 ± 2 °C. After 2 h, the excess water was removed by suspending the specimens to drain for 10 min. The mass and dimensions of the specimens were recorded followed by an additional period of soaking for 22 h. 

Thickness swelling and water absorption were reported according to Equations (2) and (3).

(2) $$\mathrm{Thickness}\;\mathrm{swelling}\;\left(\%\right)=\frac{\mathrm{Thickness}\;\left(\text{after soaking}\right)-\mathrm{Thickness}\;\left(\text{before soaking}\right)}{\mathrm{Thickness}\;\left(\text{before soaking}\right)}\times 100$$

(3) $$\mathrm{Water}\;\mathrm{absorption}\;\left(\%\right)=\frac{\mathrm{Mass}\;\left(\text{after soaking}\right)-\mathrm{Mass}\;\left(\text{before soaking}\right)}{\mathrm{Mass}\;\left(\text{before soaking}\right)}\times 100$$

**Figure 2:**
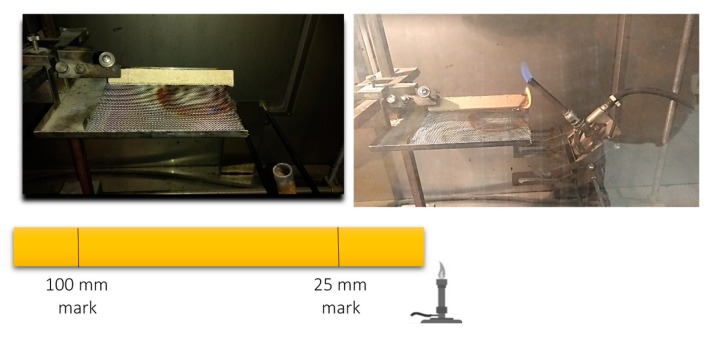
Burning test set-up in the enclosed laboratory hood and test specimen.

Burning and flammability characteristics of the specimens were tested according to ASTM D635 [30]. This test is a small-scale laboratory screening procedure to compare the extent of burning between samples. In this test, a bar specimen supported horizontally at one end was subjected to a flame at a 45° angle for 30 s in an enclosed laboratory hood, as shown in Figure 2. The specimens were 125 mm long by 13 mm wide, and 9.5 mm thick. The specimens were conditioned for 48 h at a relative humidity of 53% ± 2% and temperature of 23 ± 2 °C prior to testing. Two reference marks were placed on each specimen at 25 mm and 100 mm distance from the free end. 

To determine the relative amount of drained starch during contact dewatering in the presence of fire retardant, a Beckman DU7500 UV (Brea, CA, USA) spectrophotometer was used. The filtrate from the contact dewatering was kept from the panels prepared with formulations containing starch and boric acid/borax. The filtrate of a control sample (not containing boric acid/borax) was also tested. One mL of 12% *v*/*v* iodine-potassium iodide (I_2_–KI) solution was added to 8 mL of filtrate (from dewatering). Once the I_2_–KI was added, a blue-colored complex from the starch/I_2_–KI was formed immediately. The absorbance of the color intensity at 620 nm was determined using the UV spectrophotometer [31].

Significant differences between the independent variables were determined by performing a two-way analysis of variance (ANOVA) at the 95% confidence level. The two-way ANOVA was followed by Duncan’s Multiple Range Test (DMRT) as a post hoc test. The two tests were performed using IBM SPSS Statistics software version 25.

## 3. Results and Discussions

### 3.1. Morphology and Surface Characterization

**Figure 3:**
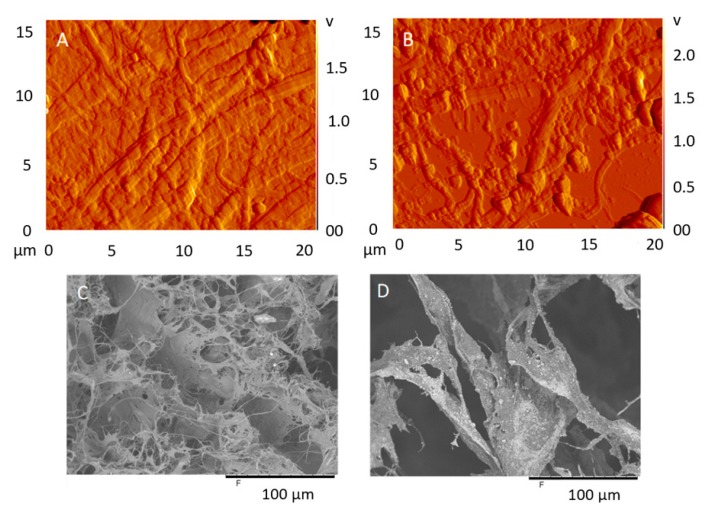
(**A**) AFM image of CNF, (**B**) AFM image of FiberLean^TM^, (**C**) SEM image of CNF, and (**D**) SEM image of FiberLean^TM^.

In order to examine the morphology of the binders (CNF and FiberLean^TM^), SEM and AFM were used. The SEM images (Figure 3C,D) showed a higher degree of fibrillation in the CNF, as opposed to FiberLean^TM^ observed from the increased fraction of the fine elements. One can also notice the deposition of the calcium carbonate particles on the microfibrillated cellulose of FiberLean^TM^. The AFM image in Figure 3A also showed the fine elements of CNF. In the FiberLean^TM^ image (Figure 3B), calcium carbonate particles are seen scattered around the cellulose microfibers, and they appear to vary in size from 1 to 2 μm for the larger particles. The surface zeta potential (ζ) showed that FiberLean^TM^ exhibited a mean negative potential of −18.2 mV, which was lower than that of CNF (−22.2 mV). The surface charge of pure calcium carbonate particles is −14.5 mV [32]. It is likely that the decrease in the mean negative value of FiberLean^TM^ as opposed to CNF is due to the reduction in the exposed area of CNF.

The indirect measurement of particle size revealed that the median volume distribution (D50) of FiberLean^TM^ was approximately twice as much as that of CNF (141 vs. 65 µm). These two values are considerably larger than the fibrils’ diameter shown in Figure 3A,D which suggests potential agglomerations in the tested samples. 

### 3.2. Mechanical Characterization of the Panels

**Figure 4:**
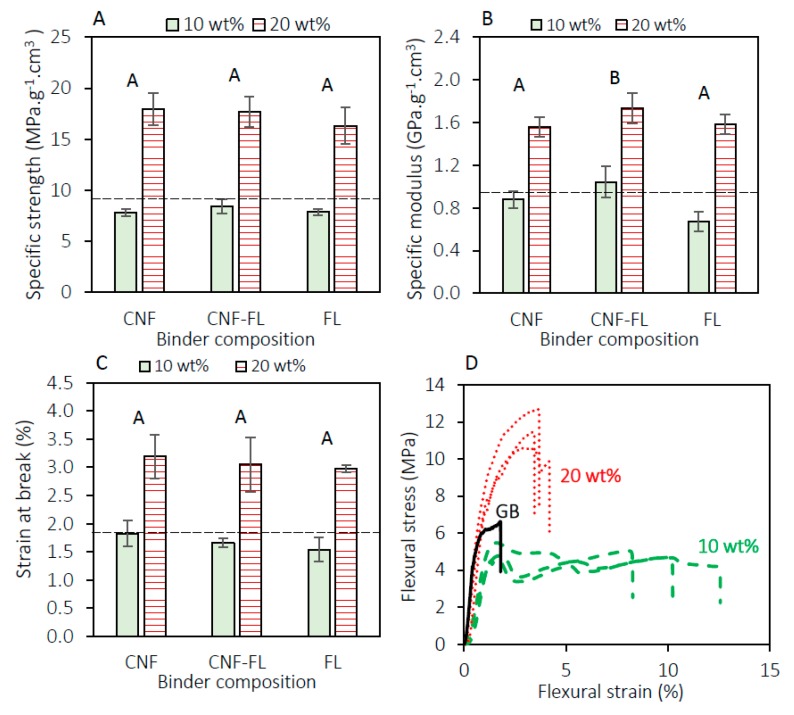
Effect of binder dosage and binder type on (**A**) specific strength, (**B**) specific modulus, (**C**) strain at break, and (**D**) stress vs. strain curve. The dashed horizontal line shows the corresponding value of commercial gypsum board. Columns with common letters are not statistically different at the 95% confidence level. (FL: FiberLean^TM^).

Figure 4 shows the effect of two binder levels (10% and 20% by weight) on the specific strength, specific modulus, and strain at break of the panels (density approximately 0.6 g/cm^3^). The figure also shows the differences between the three binder compositions (CNF, CNF-FiberLean^TM^ 50:50 and FiberLean^TM^). There was a significant difference between the 10 wt.% and 20 wt.% binder for all the flexural properties. The specific strength results have doubled from an average of 8 MPa·g^−1^·cm^3^ for panels prepared with 10 wt.% binder to 17 MPa·g^−1^·cm^3^ for the 20 wt.%-binder panels. The two-way ANOVA also revealed that there was no significant difference between the three types of binders at a 95% confidence level. Commercial gypsum board, used as a reference, exhibited a specific strength of 9.4 MPa·g^−1^·cm^3^. The statistical test showed that not all the means of the tested binders were the same for the specific modulus. Post-hoc analysis revealed that the specific modulus for panels prepared with the CNF-FiberLean^TM^ binder was significantly higher than the other two binders. Similar to specific strength, there was a significant difference in the specific modulus results between the 10 wt.% and the 20 wt.% binder. In the strain at break figure, no significant difference was observed among the three types of binders. However, a higher level of the binder (20 wt.%) has almost doubled the strain at break of the panels (1.7% vs. 3.1%). The stress vs. strain figure shows that the panel bonded with a 20 wt.% binder failed at once (panel core and paper laminae). Interestingly, the panels prepared with the 10 wt.% binder showed a different behavior. The core of the panel failed first (strain at break ~1.7%) followed by the failure of the paper laminae at a strain at break of approximately 10%. 

The adhesion properties of cellulose nanofibrils have been examined in light of several theories [8]. We believe that hydrogen bonding and the web-like network of CNF, that provide mechanical support around the wood particles, are likely explanations of the remarkable mechanical properties of the CNF-bonded panels. The substantially high surface area of CNF enhances the hydrogen bonding interaction between CNF and the wood particles. In addition, the web-like structure of CNF provides a mechanical support between wood particles, leading to an improved strength of the panels.

This first set of mechanical experimentation also highlighted the usefulness of paper lamination on the top and bottom surfaces of the core panel. In a previous study, Amini et al. (2017) [3] found that particleboard prepared with 20 wt.% CNF binder and without paper laminate resulted in a specific strength of 9.4 MPa·g^−1^·cm^3^. It is believed that the paper surfaces absorbed a large amount of energy during the flexural test, leading to an increase in the load-carrying capacity [33], as opposed to the ‘unlaminated’ particleboard.

**Figure 5:**
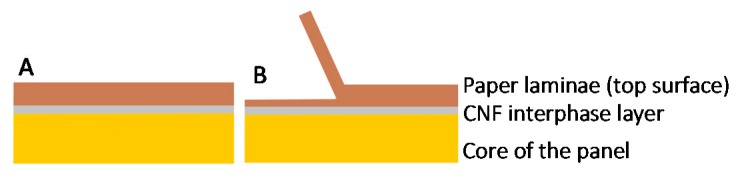
Schematic representation of (**A**) the assembly of the core, interphase and top surface of the paper laminae, and (**B**) mode of failure occurring within the paper laminae.

The CNF interphase layer was critical for maintaining a desirable adhesion between the paper and the core. The failure when peeling the paper laminate in the z-direction occurred within the paper thickness (Figure 5). This indicates that the bond strengths between the interphase (CNF) and both the core and the paper were larger than the bond strength within the paper substrates. This mode of failure is the desirable mode in gypsum board [34]. We are of the opinion that the densification during hot-pressing led to the interpenetration of CNF [8,17,35] into the core and the paper. Another interesting observation from the flexural test specimens was that the panels bonded with 10 wt.%-binder level showed a shear failure in the middle of the specimen. This failure was not observed in the panels bonded with the 20%-binder level. The CNF became increasingly available in the 20%-binder panels, allowing a stronger bonding in the core. To assess this, the internal bond test (z-direction tensile test) was conducted on panels formed with the two binder levels (10 wt.% and 20 wt.%). The test results showed that panels containing 10 wt.% binder had an internal bond of 0.17 MPa, with failure occurring in the middle of the specimens. This value exceeds the standard requirement indicated by the American National Standards Institute [36]. The results of the second group of panels (produced with 20 wt.% binder) are not reported herein because of the unacceptable failure occurring between the hot-melt adhesive and the metal block in all the specimens. In other words, the internal bond in the 20 wt.%-binder panels exceeded the bond strength between the hot-melt adhesive and the metal block (which was 0.24 MPa). This also means that the bond strength in the 20 wt.%-binder panels was higher than that in the 10 wt.%-binder panels (>0.24 MPa versus 0.17 MPa). This difference in internal bond resulting from the increased level of CNF could explain the absence of shear failure in the tested specimens.

**Figure 6:**
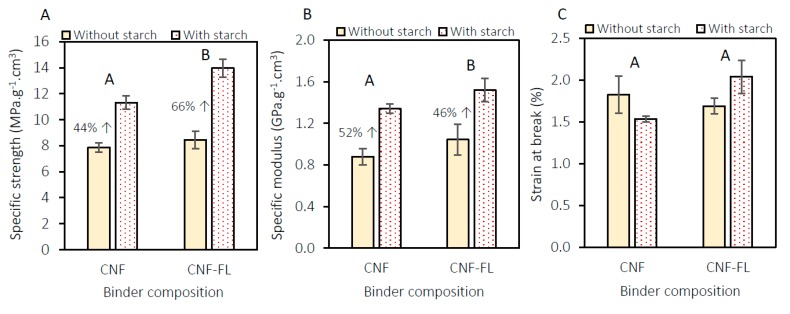

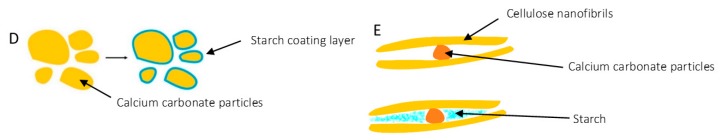
Effect of starch and binder composition (at 10 wt.% load) on (**A**) specific strength, (**B**) specific modulus, and (**C**) strain at break. (**D**) schematic representation of calcium carbonate particles before and after coating with starch, and (**E**) starch filling in the gaps surrounding the calcium carbonate particles.

To further enhance the flexural strength at a low binder level, we examined the addition of modified ‘ethylated’ starch (Ethylex^®^ 2025) to the binder formulation. It was also hypothesized that the addition of starch would have a positive effect on the flexural properties of the panels upon the addition of boric acid/borax, as will be discussed in the forthcoming sections. As a general trend, adding starch (7.6 wt.% of the binder formulation) significantly improved the specific strength and specific modulus of the panels produced with CNF and CNF-FiberLean^TM^ 50:50. The addition of starch and the binder composition did not seem to significantly affect the strain at break of the panels. The most striking result to emerge from the data is that the addition of starch considerably favored the presence of FiberLean^TM^. In other words, the presence of calcium carbonate appeared to act synergistically with the starch, and resulted in even further improvement in the specific strength. When starch was added to the FiberLean^TM^-containing formulation, it resulted in a 66% increase in the specific strength, compared to 44% when the binder was only CNF (Figure 6A). There are two likely explanations for this result. First, the particles of calcium carbonate (in FiberLean^TM^) were coated by starch (Figure 6B) during mixing. “Uncoated” mineral particles (e.g., calcium carbonate) are known to obstruct the fiber–fiber interaction in paper products [37]. Several studies have revealed that cationic and amphoteric starch could be used to increase the fiber–fiber interaction by coating the mineral particles, hence improving paper quality [38,39,40]. The retention of the mineral was also increased as a result of adding starch during the papermaking process [41]. These data must be interpreted with caution, because the starch used in the published studies was an ionic starch (i.e., bearing a positive or negative charge), while the starch used herein is an ethylated starch (i.e., not bearing a charge). Despite the non-ionic nature of the starch used in this study, it performed synergistically with the calcium carbonate similar to the ionic starch, and resulted in increased strength.

The second explanation is that the starch fills the gaps between the wood particles and fibrils (Figure 6C). The presence of calcium carbonate particles could create points of stress concentration [39] within the panel structure. Filling the voids surrounding those points could help to reduce stress at these locations.

**Figure 7:**
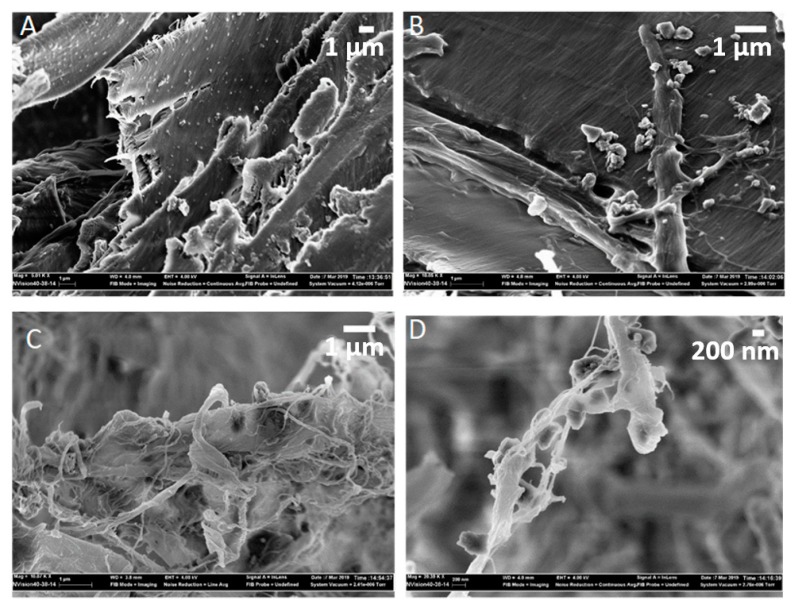
SEM of (**A**,**C**) fractured surfaces of panel samples without starch at a magnification of ×5000 and ×10,000, respectively, (**B**,**D**) panel samples with starch at a magnification of ×10,000 and ×20,000, respectively.

The above discussion appeared to be substantiated by the SEM of the fractured surfaces of the panels. Without starch, the calcium carbonate particles were found scattered on wood particles (Figure 7A) in multiple locations. In the same sample, the calcium carbonate particles were seldom found on the surface of CNF (Figure 7C). In the presence of starch, SEM revealed the attachment of calcium carbonate particles on the surface of CNF, as opposed to wood particles (Figure 7B). This feature was absent in the samples without starch. Figure 7D shows a close-up image of calcium carbonate particles attached to CNF. It can thus be assumed that the scattering of “uncoated” calcium carbonate particles on wood particles could obstruct the bonding between the particles and CNF, hence reducing the flexural strength. Referring back to the SEM image in Figure 3D, calcium carbonate particles were found scattered on the microfibrillated cellulose. This is opposite to what was observed in Figure 7A,C. A possible explanation for this discrepancy is that in the pure FiberLean^TM^ (Figure 3D), the only surrounding medium was the microfibrillated cellulose. When wood particles were present in the system, the calcium carbonate favored them over the microfibrillated cellulose or the CNF. When starch was added, the pattern was shifted back towards attachment to CNF (or microfibrillated cellulose), but not in a similar pattern. With starch, calcium carbonate particles were found on a ‘dangling’ bundle of CNF between wood particles (or possibly between other fibrils). This could indicate that the calcium carbonate particles are not attached (or deposited) on the CNF (likely due to van der Waals interactions) as in Figure 3D, and the attachment is rather due to hydrogen bonding between ‘coated’ calcium carbonate particles and cellulose nano- or microfibrils.

Furthermore, one could also notice the larger size of calcium carbonate particles in Figure 7B,D compared to those observed in Figure 7A. This is indicative that starch could potentially lead to aggregations of calcium carbonate particles, which could be a reason that those particles selectively adhere to wood and CNF.

**Figure 8:**
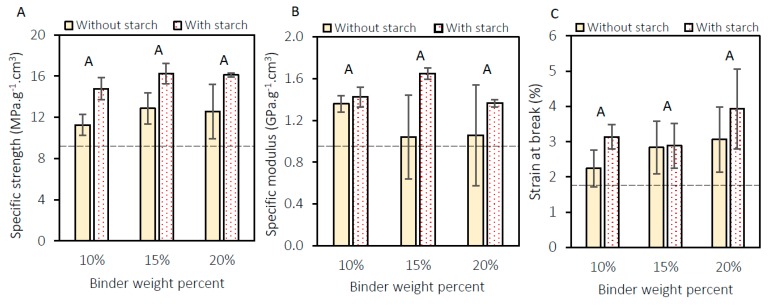
(**A**) Specific strength, (**B**) specific modulus and (**C**) strain at the break of panels with a density of 0.5–0.52 g/cm^3^. The dashed horizontal line shows the corresponding value of commercial gypsum board. Columns with common letters are not statistically different at a 95% confidence level.

The previous results have strengthened our confidence that CNF-FiberLean^TM^ 50:50 with starch is suitable to produce high-quality panels. The flexural properties were subsequently evaluated for panels with a density of approximately 0.5 g/cm^3^. Figure 8 shows the specific strengths, specific modulus, and strain at break of panels made of three wood particles to binder ratios in the presence or absence of starch.

The results support our hypothesis that starch is important in enhancing the strength and also the modulus of low-density panels (~0.5 g/cm^3^). The two-way ANOVA test showed that the addition of starch significantly improved the specific strength and modulus of the panel, while no significant difference was observed between the binder types. However, both starch and binder type did not have any significant effect on the strain at break. Without starch, the average specific strength of the panels was 12 MPa·g^−1^·cm^3^, whereas the average specific strength of the panels with starch was approximately 16 MPa·g^−1^·cm^3^. The average specific moduli in the absence and presence of starch were 1.2 GPa·g^−1^·cm^3^ and 1.5 GPa·g^−1^·cm^3^, respectively. It appears that the addition of starch is necessary in order for the low-density panels to perform better than gypsum board.

### 3.3. Thickness Swelling and Water Absorption

**Figure 9:**
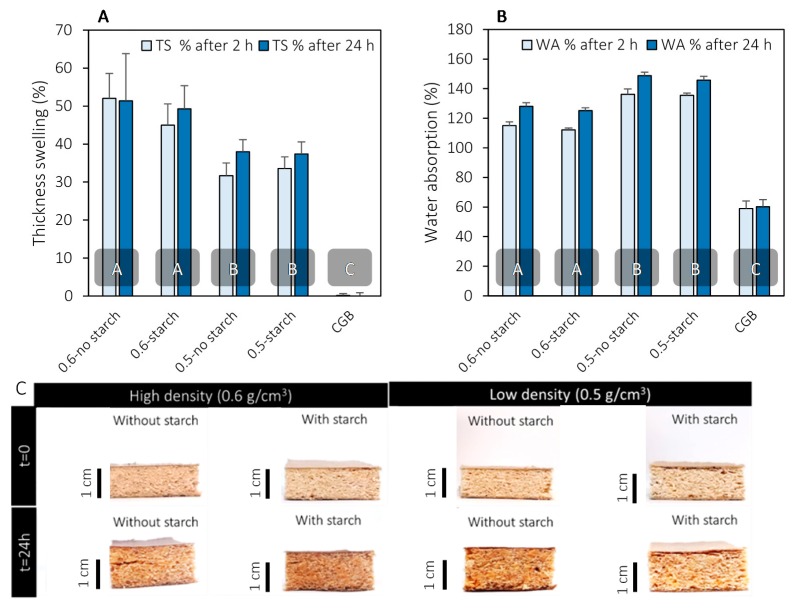
(**A**) thickness swelling of low and high-density panels and comparison with commercial gypsum board (CGB), (**B**) water absorption of low and high-density panels and comparison with this same CGB, and (**C**) sample images at time zero and at the end of the test (after 24 h). In Figures A and B, columns with common letters are not statistically different at the 95% confidence level.

The thickness swelling and water absorption results are shown in Figure 9. On average, gypsum boards had negligible thickness swelling (~0%). It is seen from Figure 9A that reducing the density from 0.6 to 0.5 g/cm^3^ significantly reduced the thickness swelling by approximately 40% (from 52% to 32%). The reduced thickness swelling in low-density panels is attributed to the reduction in the material per unit volume (i.e., an increase in pores volume). The addition of starch to either high- and low-density panels did not significantly affect the thickness swelling. This could suggest that starch does not occupy a large volume of the pores, and it only fills the small gaps between wood particles and CNF, hence not affecting the thickness swelling. As for the water absorption, an expected opposite trend was observed between the high- and low-density panels. Figure 9B shows that the water absorption (after 24 h) increased from 128% to 149% when the density decreased from 0.6 to 0.5 g/cm^3^ in the absence of starch. Adding starch resulted in an increase in water absorption from 125% to 146%. Statistically, the addition of starch did not seem to affect the water absorption similar to thickness swelling. After soaking for 24 h, the pieces remained intact, and no disintegration of the specimens was observed (Figure 9C).

Gypsum board exhibited a water absorption of 60%. It is important to note that these panels are intended for internal use in dry conditions. In order to expand their applications to perform under wet conditions, further treatments will be required, which is the scope of our future studies. Also, there are no standard specifications for gypsum board for thickness swelling and water absorption. The standard specification for gypsum board uses the humidified deflection test as a procedure to evaluate the deflection of gypsum board under high humidity conditions. Given the promising performance of our panels under harsh conditions (soaking for 24 h), we believe that the panels will compete with gypsum board when tested at high humidity (less harsh conditions). It is worth pointing out that the panels tested for thickness swelling and water absorption did not have boric acid/borax (fire retardant) in their composition. If there was boric acid/borax, some amount could be assumed to be washed away, but this is unlikely to affect the fire retardancy. This assumption is based on the evidence that despite the wash-away of the fire retardants that occurs during contact dewatering, the produced fire-retardant panels still exhibited a fire-retardancy comparable to that of gypsum board, as will be discussed in detail in the next section.

### 3.4. Burning Characteristics and Effect of the Fire Retardant

The panels were tested for their burning and flammability properties according to ASTM D635 [30]. After 30 s of exposure to flame, the untreated sample continued to burn beyond the 25 mm reference mark, and about 50% of the strip length was completely burnt. The calculated linear burning rate for the untreated specimens was 9.2 mm/min. When 5 wt.% boric acid/borax (1:1) was added to the formulation, a substantial enhancement in the burning characteristics of the tested specimens was observed. A previous study showed that a 1:1 ratio of boric acid and borax successfully imparted fire-retardancy to fiberboards [42,43].

**Figure 10:**
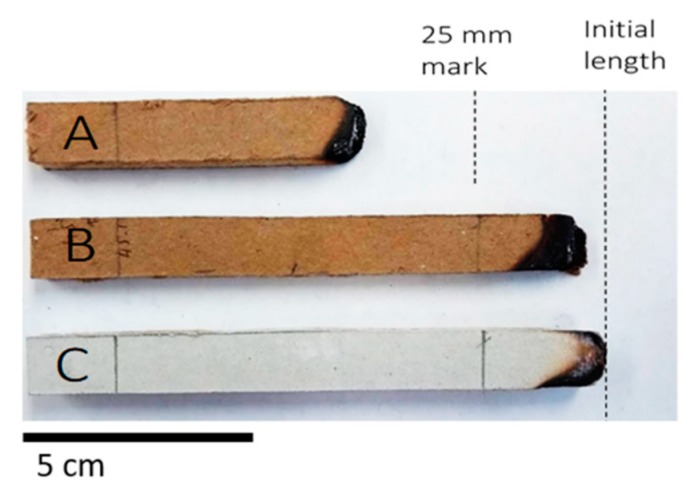
Burning characteristics of (**A**) untreated, (**B**) treated with 5% boric acid/borax, and (**C**) commercial gypsum board.

As seen in Figure 10, the flame front in the treated sample did not travel beyond the 25-mm reference mark. This is similar to the behavior of gypsum board strips under the same conditions, which is known for its excellent fire resistance. In such a case, the linear burning rate was not reported for the treated samples and for gypsum board according to the standard. There is a general agreement that boric acid forms a coating or protective layer on the surface of wood particle at high temperature [44,45,46]. Another proposed theory is that, at 100–300 °C, boric acid may catalyze the dehydration and other oxygen-eliminating reactions of wood [46]. It has also been found that the formation of charring may be enhanced through the isomerization reactions of the newly formed polymeric materials catalyzed by boric acid. We also expect that the chemically combined water in the borax is slowly released at elevated temperatures, which effectively leads to a retarding heat transfer. This latter mechanism is similar to that of gypsum board when the crystallized water bonded to the calcium sulfate is released at high temperatures. It is interesting to note that such improvement in burning characteristics is not attributed to the full amount of the added boric acid/borax (5 wt.%). 

We believe that a portion of the fire-retardant mixture was drained during the contact dewatering due to its polar nature. We also believe that a portion of the borax dissociated and covalently crosslinked [47] the starch (and CNF), leading to increased starch retention in the panel, as will be discussed in the next section.

Turning now to the mechanical properties of the panels treated with a fire retardant, it was found that the addition of 5 wt.% boric acid/borax mixture did not significantly affect the flexural properties (Table 1). To date, there has been little agreement on the effect of boric acid, borax, or both, on the mechanical properties of the wood-based panels. Generally, the addition of boric acid/borax seemed to decrease the flexural strength of particleboard. In one study [48], a low level (0.5%) of boric acid/borax (5:1) appeared to increase the flexural strength and flexural modulus of the wood composite. A higher dosage of the fire retardant (5 wt.%) decreased the flexural strength and flexural modulus significantly. The authors attributed this decrease to the deposition of the flame-retardant on the board fibers, or the degradation of cellulose fibers by the flame retardant. Pedieu et al. (2012) reported different results for internal bond and flexural strength [49]. They found that the addition of 16% boric acid to the particleboard increased the internal bond, while a 12% fire retardant resulted in a decrease in flexural strength. The increase in internal bond was attributed to the reduced pH, which promoted the curing of the urea-formaldehyde resin. The decrease in flexural strength, however, was attributed to the decrease in the overlapping effect of fine wood particles (used in the particleboard surface), due to the granular shape of boric acid. The authors also believed that the pre-cure of urea formaldehyde resin in the surface layer (due to the high acidity) could play a role in the reduction in flexural strength. We should, however, sound a note of caution with regard to comparing such findings. In these previous findings, the process of making particleboard was a dry-based process. In contrast, our system comprised up to 80% water.

**Figure 11:**
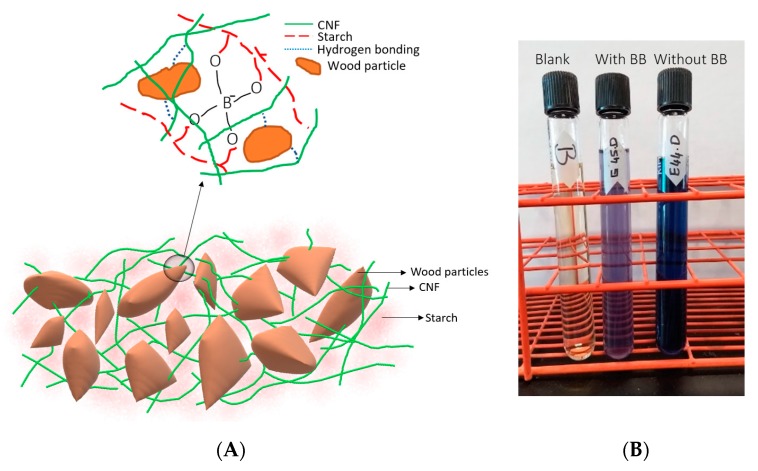
(**A**) Schematic representation of the interactions between the various components of the panel and (**B**) Color intensity after adding the iodine-potassium iodide to the filtrate for treated and untreated samples. (BB: Boric acid and borax mixture).

As previously mentioned, boric acid/borax did not decrease the flexural strength despite its previously found effect in reducing this mechanical attribute. In our system, the addition of boric acid/borax is expected to covalently crosslink [47] the starch, leading to an increase in its viscosity, which consequently increases the starch retention. In other words, the amount of drained starch is less in the sample treated with boric acid/borax than the untreated sample. In previous studies, boric acid has been found successful in the crosslinking of starch and polyvinyl alcohol (OH-containing components) [50,51]. When iodine–potassium iodide (I_2_–KI) solution was added to the filtrate (water drained from dewatering), it formed a blue-colored starch/I_2_–KI complex. Figure 11A shows a schematic representation of the proposed interactions between the various components of the panel.

The color intensity of this complex was measured by a UV-vis spectrophotometry, which gave a relative indication of the amount of starch in the filtrate. At 620 nm wavelength (Table 1), the absorbance value of the sample containing boric acid/borax, was almost half the untreated sample, as shown in the middle tube with a lighter color solution in Figure 11B. This indicated that the ‘retained’ amount of starch in the treated sample (with boric acid/borax) was almost twice that of the untreated sample.

**Figure 12:**
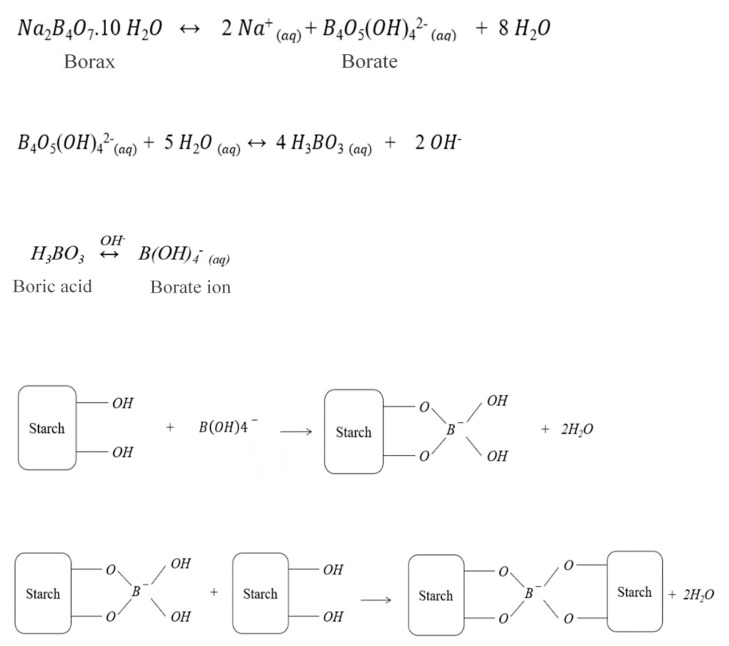
The dissociation of borax in water and the proposed mechanism between boric acid/borax and starch.

The proposed mechanism [50,52] is through the dissociation of borax (Na_2_B_4_O_7_) in water (Figure 12). The outcome of the dissociation reaction is borate ions and water molecules. Borate ions further react with water, giving boric acid and hydroxide ions. In the presence of the hydroxide ions, the additional boric acid is converted to borate ions. The borate ions subsequently interact with OH-containing materials, such as starch and CNF, leading to a reduction in the drained starch, as confirmed by a UV-spectrophotometer. Despite the crosslinking of the starch and possibly the CNF, the flexural strength did not significantly increase. This is possibly due to the fact that not all the borax contributed to the crosslinking. It is believed that part of the borax was washed away, and the other part contributed to the fire retardancy of the panel. The positive effect, however, lies in maintaining the same flexural strength different from the previous findings, which showed that the addition of boric acid reduced the flexural strength.

## 4. Conclusions

Wallboard laminated panels were successfully with high strength and fire retardancy. At high and low density, the addition of starch was imperative in improving the flexural properties of the panels, especially in the presence of FiberLean^TM^. Reducing the density of the panels decreased the thickness swelling. Treating the panels with 5% boric acid/borax (1:1) was effective in imparting fire-resistant properties to the panels similar to commercial gypsum board. Also, the addition of the fire retardant did not affect the flexural properties negatively, as previously reported in the literature. It was found that the boric acid/borax mixture also increased the retained starch in the system confirmed by the relative assessment of starch in the filtrate. It is presumed that this increase in the retention is a result of the crosslinking of starch by boric acid/borax mixture. We believe that this research will pave the way for further advancement in green building applications and for the commercialization of the use of cellulose nanofibrils in binders application.

## Figures and Tables

**Table 1:** Flexural properties of treated and untreated panels and the UV absorbance value for the two panels.

Treatment	Specific Strength (MPa·g^−1^·cm^3^)	Specific Modulus (GPa·g^−1^·cm^3^)	Strain at Break (%)	Absorbance at 620 nm
Without fire retardant	16.0 ± 1.0	1.3 ± 0.2	3.9 ± 1.9	1.59
With fire retardant	16.8 ± 0.8	1.6 ± 0.2	3.4 ± 1.0	0.86
